# A rare presentation of subphrenic abscess caused by *Proteus mirabilis* in a non-immunocompromised patient: A case report

**DOI:** 10.3892/mi.2025.249

**Published:** 2025-06-17

**Authors:** Lasha Chkhikvadze, Elisabed Chikobava, Nino Kantaria

**Affiliations:** 1 American MD Program, Tbilisi State Medical University, 0186 Tbilisi, Georgia; 2 Department of Internal Medicine, New Hospitals, 0162 Tbilisi, Georgia

**Keywords:** subphrenic abscess, *Proteus mirabilis*, multidrug resistance, percutaneous drainage, intra-abdominal infections

## Abstract

Subphrenic abscesses are rare, yet clinically significant, and are often associated with intra-abdominal infections. The present case report describes the case of a 77-year-old male patient with an unusual presentation of a subphrenic abscess caused by *Proteus mirabilis* without typical predisposing factors. The patient experienced a prolonged 2-year course characterized by recurrent febrile episodes and respiratory symptoms. The diagnosis was confirmed through imaging and microbiological culture, with percutaneous drainage and culture-directed antibiotic therapy, including meropenem, leading to successful treatment. The multidrug-resistant nature of the pathogen, coupled with the atypical presentation of the patient, posed significant challenges in both diagnosis and management. The case described herein underscores the importance of comprehensive diagnostic imaging, timely intervention and individualized treatment strategies in managing such complex infections.

## Introduction

Subphrenic abscesses are collections of infected fluid located beneath the diaphragm, typically in the space between the diaphragm and the upper abdominal organs. These abscesses are anatomically bordered inferiorly by the transverse colon, mesocolon and greater omentum (1). Although they are relatively uncommon, subphrenic abscesses are clinically significant and often arise as complications of intra-abdominal infections following surgery, trauma or immunosuppression (2). When typical risk factors are absent, the diagnosis and management of subphrenic abscesses can be more complex.

The causative pathogens differ by the type of surgery, with *Staphylococcus aureus* infection being common following gastric procedures and *Bacteroides fragilis* and *Clostridium* species infections being more common following colon surgery or appendicitis (2). However, *Proteus mirabilis* (*P. mirabilis*), a Gram-negative bacterium typically associated with urinary tract infections, is a rare cause of subphrenic abscesses and is particularly uncommon among immunocompetent patients.

The management of subphrenic abscesses requires a tailored approach, particularly when atypical or drug-resistant pathogens are involved. The present study describes a rare case of an immunocompetent patient with subphrenic abscess caused by *P. mirabilis*, without the usual risk factors (3). The prolonged 2-year course and multidrug-resistant nature of the infection made both diagnosis and treatment particularly challenging.

The case presented herein underscores the importance of comprehensive diagnostic imaging and culture-directed antibiotic therapy in the management of such infections. It further highlights the critical role of timely intervention, including percutaneous drainage and the judicious use of antibiotics, to achieve favorable clinical outcomes in cases involving rare and resistant pathogens.

## Case report

A 77-year-old male patient presented for an outpatient evaluation by an internist at New Hospitals (Tbilisi, Georgia), with a 2-year history of recurrent febrile episodes, with temperatures ranging from 37 to 39˚C, partially alleviated by acetaminophen and ibuprofen. The fever was accompanied by generalized weakness, nausea and vertigo. At 1 month prior to the presentation, the patient developed a nocturnal spasmodic cough producing white sputum.

His medical history was notable for an episode of community-acquired pneumonia 2 years prior, complicated by pleuritis and a right-sided pleural effusion. The pleural fluid was exudative, with an adenosine deaminase level of 56 U/l. No specific infectious agent was identified as a cause of pneumonia. Tuberculosis was ruled out with interferon gamma release assay. The condition was managed with moxifloxacin and prednisone.

The patient was also treated for a lower urinary tract infection 2 months prior. At 13 years prior to this, he had undergone surgical repair for an abdominal aortic rupture, and 5 years prior, a laparoscopic cholecystectomy had been performed.

At the time of the presentation, the vital signs of the patient were as follows: A temperature of 37.7˚C, heart rate of 65 beats per minute, blood pressure of 130/70 mmHg, respiratory rate of 22 breaths per minute and an oxygen saturation of 95% in room air. A physical examination yielded no significant findings.

Laboratory investigations demonstrated the following: A red blood cell count of 3.92x10^6^/µl, hemoglobin level of 10.4 g/dl, mean corpuscular volume of 78.6 fl, a platelet count of 354x10^3^/µl, white blood cell count of 22.09x10^3^/µl, neutrophil count of 18.83x10^3^/µl and a lymphocyte count of 1.39x10^3^/µl. Arterial blood gas analysis revealed a pH of 7.41, partial pressure of carbon dioxide level of 41 mmHg, a sodium level of 135 mmol/l, a potassium level of 4.3 mmol/l, a calcium level of 1.14 mmol/l, a glucose level of 111 mg/dl, a lactate level of 1.7 mmol/l and a bicarbonate level of 26 mmol/l.

**Figure 1:**
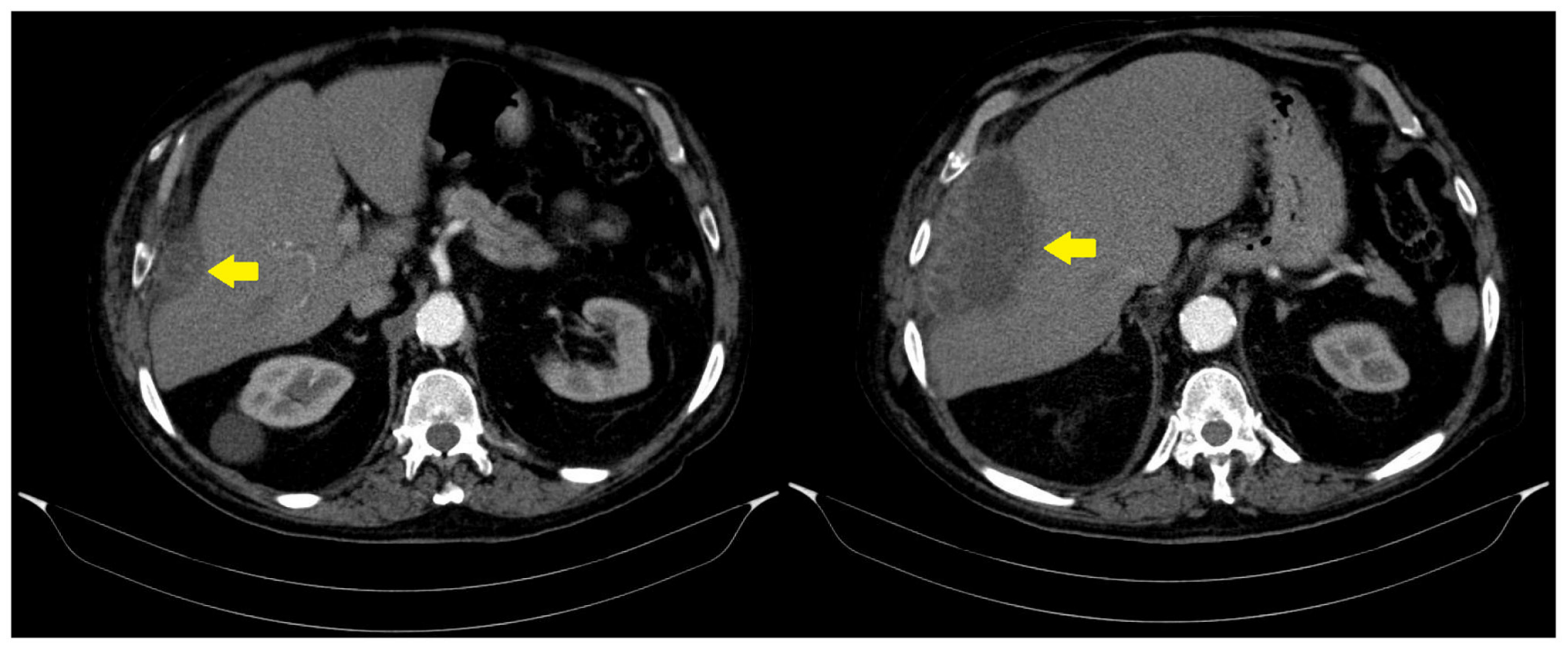
Computed tomography images illustrating a complex cystic mass measuring 11.5 cm in diameter, located in the right subdiaphragmatic region, extending into the thoracic soft tissue at the 7th and 8th intercostal spaces. The mass involved the right pleura and middle lobe of the lung, exerting compressive effects on the right liver lobe. Atelectasis was observed in the lower and middle lobes of the right lung and the lower lobe of the left lung. A small right-sided pleural effusion was also noted. The image on the left panel represents the upper axial slice, illustrating the superior aspect of the subdiaphragmatic abscess. The image on the right panel represents the lower axial slice, providing clearer visualization of the caudal extent of the abscess. Yellow arrows indicate the abscess in both images.

Given the persistent fever of unknown origin, a chest computed tomography (CT) scan was performed, which revealed a subphrenic abscess (Fig. 1). To aid in diagnosis and intervention, an 18 G puncture needle was initially used to aspirate the abscess and collect a specimen for analysis. Subsequently, a 12CH pigtail catheter was inserted to enable continuous drainage and facilitate abscess lavage with saline four times daily, following local hospital protocols.

The aspirate analysis revealed pinkish-yellow, turbid, exudative fluid with a high lactate dehydrogenase level of 678 U/l (normal range, 140-280 U/l), an elevated leukocyte count (normal value, <500 cells/µl) and a predominance of lymphocytes at 82% (normal value, <50%), while polymorphonuclear cells accounted for 18% (normal value >50%).

A specimen biopsy was obtained for microscopy and immunohistochemistry due to concerns about possible malignancy. These analyses were performed at the Pathology Laboratory of New Hospitals, and as per routine local practice, only the final report was provided without image documentation. Microscopy revealed fibrous and hyalinized stroma with the infiltration of white blood cells. Immunohistochemistry demonstrated CD3-, CD20- and PAX5-positive lymphocytes; CD38-positive plasma cells; CD68-positive macrophages; and CD15-positive granulocytes. CD30, a lymphoma marker, was negative, thereby excluding an underlying malignant etiology.

Bacteriological analysis of the aspirated fluid identified *P. mirabilis* revealed resistance to gentamicin, imipenem, cefotaxime, cefazolin, trimethoprim (TMP)/sulfamethoxazole (SMX), amoxicillin/clavulanate and tigecycline based on phenotypic testing, while it revealed sensitivity to fluoroquinolones, tobramycin, amikacin, meropenem, ceftazidime, cefepime, piperacillin-tazobactam. Genotypic resistance testing [e.g., for extended-spectrum β-lactamase (ESBL) or carbapenemase genes] was not performed. Immunohistochemical analysis revealed inflammation without any evidence of malignancy.

During hospitalization, the patient was commenced on empirical treatment with IV meropenem, administered at 2 g every 8 h for 2 weeks. Following the antibiotic sensitivity report, which revealed susceptibility to fluoroquinolones, the treatment was adjusted to IV moxifloxacin, at 400 mg once daily, for the following 6 weeks. This de-escalation was guided by antimicrobial stewardship principles to reduce unnecessary carbapenem use, once effective narrower-spectrum therapy was identified. Moxifloxacin was selected due to its confirmed activity against the isolated *P. mirabilis* strain and its excellent intra-abdominal tissue penetration, which is essential for treating deep-seated infections, such as subphrenic abscesses (4). Maintaining IV administration ensured consistent therapeutic levels in this elderly patient during prolonged treatment.

The patient was closely monitored through regular follow-up visits over a 6-month period. He remained afebrile and symptom-free throughout this period, with no recurrence of abdominal pain, respiratory symptoms, or signs of systemic infection. Serial laboratory investigations, including analyses of complete blood count and C-reactive protein levels, yielded results which were within the normal limits. A follow-up abdominal ultrasound performed at 3 and 6 months post-discharge revealed the complete resolution of the abscess, with no residual fluid collection or new abnormalities. As per local clinical practice, ultrasound images were not archived or provided to the referring physician. The patient did not require further antibiotic therapy and returned to his usual daily activities without restrictions. No late complications or relapses were reported.

## Discussion

A subphrenic abscess, also referred to as a subdiaphragmatic or infra-diaphragmatic abscess, is characterized by a localized collection of pus in the subphrenic space. Although rare, its exact incidence remains unclear. The condition is typically unilateral, most often occurring on the right side in ~50% of cases, with 40% on the left side and 25% presenting bilaterally (1-3,5). In rare instances, both intraperitoneal and extraperitoneal involvement may be observed, as was the case of the patient described herein, who presented with a right-sided subphrenic abscess exhibiting both forms of involvement (5).

Subphrenic abscesses most commonly arise from the introduction of bacteria into the subphrenic space. Secondary subphrenic abscesses are most frequently associated with gastric and biliary surgeries, accounting for 52% of cases. Appendicitis is responsible for 8%, while colonic surgery and trauma contribute to 19 and 8%, respectively (6). Localized inflammation in the space between the liver, intestines, and lungs can also serve as a cause of subphrenic abscess (2). The diagnosis is often delayed or missed when the abscess is not related to surgery. The majority cases appear within weeks of abdominal surgery, though some may present as late as 5 months post-operatively (7). Although the patient in the present study had a history of intra-abdominal surgeries, including abdominal aortic aneurysm repair 13 years prior and laparoscopic cholecystectomy 5 years prior, the timing of these procedures did not coincide with the onset of symptoms, making them unlikely contributors to the subphrenic abscess. The source of the *P. mirabilis* infection remains uncertain. However, it is plausible that a prior episode of pneumonia, complicated by a right-sided pleural effusion ~2 years prior, played a role. Despite the absence of pathogen isolation from the pleural fluid at that time, the anatomical proximity between the pleural cavity and subphrenic space suggests the possibility of transdiaphragmatic spread. Additionally, while *P. mirabilis* is not typically associated with subphrenic abscesses, it has been documented as a causative agent in various deep-seated infections in susceptible individuals, which may support its role as a potential source in this case (8-14).

While transdiaphragmatic extension from the prior pleural effusion remains the most plausible explanation, other potential routes of infection were also considered. These include hematogenous spread, gastrointestinal translocation, or reactivation from previous abdominal surgery. However, the patient had no evidence of bacteremia, gastrointestinal symptoms, or recent surgical intervention to support these mechanisms. Therefore, the precise route of *P. mirabilis* entry remains speculative. Although a causal link remains unclear, this assumption should be viewed in light of diagnostic limitations during the earlier pneumonia episode. In Georgia, routine microbiological testing, such as bronchoalveolar lavage or pleural fluid culture, is not commonly performed for community-acquired pneumonia unless the case is severe or atypical. As a result, no causative organism was identified at that time. The pleural effusion was unilateral and exudative, making alternative causes such as cardiac origin less likely. These limitations reduce diagnostic certainty and are common in resource-limited settings, where comprehensive microbial workup is not always feasible. Nevertheless, the timing, anatomical proximity, and clinical context make a transdiaphragmatic infectious process a reasonable consideration.

Previous studies indicate that subphrenic abscesses are often polymicrobial, with aerobic bacteria, such as *Escherichia coli*, *Enterococcus spp.*, *Enterobacter* and *Staphylococcus aureus* being the predominant isolates. The most common anaerobes include *Peptostreptococcus*, *Bacteroides fragilis*, *Clostridium spp.* and *Prevotella*. Following biliary surgery, *Enterococcus* group D infection predominates, while infections with *Fusobacterium* and *Prevotella* species are common following gastric or duodenal surgery (15). Notably, the case stands out due to the isolation of *P. mirabilis* from the abscess aspirate, a rare finding in subphrenic abscesses.

*P. mirabilis* belongs to the *Proteus* genus, which includes five named species (*P. mirabilis*, *Proteus penneri*, *Proteus vulgaris*, *Proteusmyxofaciens* and *Proteus hauseri*) and several unnamed (*Proteus* 4,5,6) genomospecies (16). Although *P. mirabilis* is a common Gram-negative pathogen in clinical settings, it is not typically associated with severe infections and is more frequently found in colonizing wounds (17). However, in immunocompromised individuals, it may cause systemic infections, including urinary tract infections, biliary infections, wound infections and peritonitis (17). While *P. vulgaris* is commonly identified as a causative agent of visceral intra-abdominal abscesses, *P. mirabilis* has been reported as a pathogen in abscesses of the intracranial region, lungs, kidneys and liver, breast, iliopsoas muscle (8-14). However, there is a paucity of detailed reports on *P. mirabilis* in subphrenic abscesses, with the majority of reports focusing on its prevalence rather than its precise role or characteristics, which renders the present case report particularly significant (6,8,10,11,13,17,18). Moreover, the patient described herein immunocompetent and had no history of nephrolithiasis, which is commonly associated with *P. mirabilis*, raising questions about how the pathogen contributed to the abscess.

The present case report is notable when compared to other documented instances of *P. mirabilis* causing abscesses in atypical locations. For example, *P. mirabilis* was previously reported in a 17-year-old patient with an intracranial abscess, causing rare neurological symptoms, such as seizures and altered sensorium in an immunocompetent patient (9). Similarly, *P. mirabilis* was previously identified in a breast abscess in a 56-year-old female patient, which is unusual, as breast abscesses are typically caused by *Staphylococcus aureus* and *Streptococcus* spp (8). Additionally, *P. mirabilis* has been found in iliopsoas muscle abscesses, a site usually affected by *Escherichia coli* or *Staphylococcus aureus*, further highlighting the atypical role of this pathogen in deep-seated infections (11). In neonatal cases, *P. mirabilis* has even been implicated in brain abscesses, an uncommon occurrence given that such infections are typically caused by *Streptococcus* and *Staphylococcus* species (10).

By contrast, subphrenic abscesses are often polymicrobial, and the isolation of *P. mirabilis* as the sole pathogen, as in the case in the present study, is highly unusual, particularly in an immunocompetent patient without the typical predisposing factors, such as recent surgery, urinary tract infection, or nephrolithiasis (15). Therefore, the present case report adds valuable insight into the understanding of *P. mirabilis* and its unusual involvement in abscesses outside its typical pathogenetic scope, particularly in the absence of common predisposing conditions. The case described herein also underscores the need to consider this pathogen in the differential diagnosis of deep-seated infections, even in patients with no notable past medical history.

Typically, *P. mirabilis* is intrinsically resistant to nitrofurantoin and tetracycline, while remaining susceptible to β-lactams, aminoglycosides, fluoroquinolones and trimethoprim-sulfamethoxazole. However, in this instance, the isolated strain exhibited multidrug resistance, including resistance to gentamicin, imipenem, cefotaxime, cefazolin, TMP/SMX, amoxicillin/clavulanate and tigecycline. This posed a significant challenge in selecting an effective antibiotic regimen, as multidrug-resistant *P. mirabilis* infections complicate the management of deep-seated infections such as subphrenic abscesses (16,17). The resistance of *P. mirabilis* to β-lactams, aminoglycosides and quinolones has been increasingly reported in clinical isolates, raising concerns about the effectiveness of these agents in treating infections caused by an ESBL-producing strain (18). As a result, carbapenems, such as meropenem, have become the drug of choice for empirical therapy, particularly when resistant strains are suspected (19). In the case in the present study, meropenem was initiated as an empiric therapy for the subphrenic abscess, and the isolate was found to be sensitive to it, allowing for effective initial management and resolution of the infection. This highlights the continued relevance of carbapenems as a critical therapeutic option, despite the growing resistance patterns seen in P. mirabilis (20). However, the increasing frequency of resistance to key antibiotics, including fluoroquinolones, aminoglycosides and β-lactams, underscores the need for alternative therapeutic approaches, particularly in patients with infections caused by multidrug-resistant strains.

The clinical presentation of a subphrenic abscess can vary depending on its anatomical location. Symptoms often include fever, pain in the upper abdomen or shoulder, costal margin tenderness, abdominal tenderness and dyspnea (2). Additionally, it may present with cough, hiccups, or unexplained pulmonary manifestations such as pneumonia, pleural effusion, or basal atelectasis (2), as observed in the patient in the present study. Fever of unknown origin is also a common symptom (2).

If not treated promptly, patients may develop systemic inflammatory response syndrome, characterized by tachycardia, hypotension and oliguria, which can ultimately progress to multiorgan failure and mortality (2). Notably, the patient in the present study appeared to have had this condition for ~2 years without developing such severe complications, renderings this case particularly unusual. This prolonged clinical course raises the question of why the abscess remained undiagnosed for such a long period of time. During this period, the patient experienced intermittent febrile episodes; however, no cross-sectional imaging was performed, as he declined CT scans due to personal reluctance. This hindered early diagnostic evaluation and contributed to a delayed identification of the evolving deep-seated pathology. The subphrenic abscess was only detected after clinical deterioration prompted urgent imaging.

Laboratory findings typically include leukocytosis with neutrophilia and an elevated erythrocyte sedimentation rate (2). Blood gas analysis may initially reveal respiratory alkalosis due to hyperventilation, which can progress to metabolic acidosis if left untreated (2). Blood cultures showing polymicrobial growth are highly suggestive of subphrenic abscess (2).

Imaging plays a pivotal role in diagnosing subphrenic abscesses and guiding management decisions. Chest X-rays may reveal diaphragmatic elevation, pleural effusion, or lung abnormalities (2,21). In the case described herein, a chest X-ray performed 7 months prior incidentally revealed a small right-sided pleural effusion. The development of pleural effusion in association with a subphrenic abscess is considered to result from diaphragmatic inflammation, increasing capillary permeability and leading to the accumulation of pleural fluid.

Abdominal ultrasound is commonly used for initial detection, particularly for right-sided abscesses, due to its high sensitivity for fluid collections and real-time imaging capabilities. It aids in the assessment of abscess size and location, and is particularly useful for guiding percutaneous drainage. However, the limitations of an ultrasound include reduced efficacy for left-sided abscesses due to bowel gas interference, and its operator-dependence, meaning the quality of results can vary. Additionally, ultrasound may not be able to visualize deep or complex abscesses (22,23). When ultrasound is inconclusive or for left-sided abscesses, contrast-enhanced CT is considered the gold standard. CT provides detailed cross-sectional images that allow for precise localization of the abscess, assessment of surrounding structures, and planning of drainage procedures. It is particularly beneficial for left-sided abscesses, where ultrasound is less effective. However, CT does involve radiation exposure and may not be as readily accessible in all clinical settings (24,25). In cases of deep or challenging abscesses, endoscopic ultrasound (EUS) can be used to access difficult-to-reach areas, offering real-time imaging and minimizing the risk of intervening structures during drainage. While EUS provides a valuable alternative, it is invasive, requires specialized skills, and is not universally available (26,27).

The management of a subphrenic abscess typically involves antibiotic therapy and drainage. Empiric therapy with broad-spectrum antibiotics should be initiated immediately to cover both aerobic and anaerobic organisms. Once culture results are available, the antibiotics should be adjusted accordingly. In immunocompromised patients, antifungal coverage may also be necessary, particularly for *Candida* species (2). For drainage, percutaneous CT-guided drainage is considered the gold standard due to its high success rate, minimal invasiveness, and lower complication rates compared to surgical drainage. It allows for real-time visualization and avoids the need for general anesthesia, which is particularly beneficial for elderly patients with multiple comorbidities. CT-guided drainage is effective in controlling sepsis and can be used both for diagnostic and therapeutic purposes (28,29).

If percutaneous drainage is unsuccessful or if the abscess is loculated, surgical drainage may be required. Laparoscopic drainage is less invasive than open surgery and allows for direct visualization and drainage of the abscess. However, if laparoscopic methods fail, open surgery may be necessary, although this approach carries more risks, such as injury to surrounding organs. In severe cases, particularly in trauma or abdominal compartment syndrome, open abdomen therapy may be employed as a damage control strategy. Timely drainage and appropriate antibiotic therapy are crucial for preventing sepsis, and intensive care may be required for patients who develop multiorgan failure (30).

In conclusion, the present case report underscores the uncommon presentation of a subphrenic abscess caused by *P. mirabilis* in an immunocompetent patient without typical risk factors or a clear timeline linking the infection to prior surgical interventions. The prolonged 2-year symptom duration, the absence of severe complications, and the multidrug-resistant nature of the isolated strain posed significant diagnostic and therapeutic challenges. This highlights the critical role of comprehensive diagnostic imaging and culture-guided therapy in managing complex and atypical abscesses. Early, decisive interventions, such as percutaneous drainage combined with the strategic use of advanced antibiotics such as meropenem, proved instrumental in achieving a successful outcome. The case described herein emphasizes the need for heightened clinical awareness, meticulous treatment planning and adaptability in addressing rare, resistant infections effectively.

## Data Availability

The data generated in the present study may be requested from the corresponding author.
